# Uniconazole-induced starch accumulation in the bioenergy crop duckweed (*Landoltia punctata*) I: transcriptome analysis of the effects of uniconazole on chlorophyll and endogenous hormone biosynthesis

**DOI:** 10.1186/s13068-015-0246-7

**Published:** 2015-04-02

**Authors:** Yang Liu, Yang Fang, Mengjun Huang, Yanling Jin, Jiaolong Sun, Xiang Tao, Guohua Zhang, Kaize He, Yun Zhao, Hai Zhao

**Affiliations:** Chengdu Institute of Biology, Chinese Academy of Sciences, Chengdu, 610041 China; University of Chinese Academy of Sciences, Beijing, 100049 China; Key Laboratory of Environmental and Applied Microbiology, Chinese Academy of Sciences, Chengdu, 610041 China; Environmental Microbiology Key Laboratory of Sichuan Province, Chengdu, 610041 China; Key Laboratory of Bio-Resources and Eco-Environment, Ministry of Education, College of Life Sciences, Sichuan University, Chengdu, 610064 China

**Keywords:** Uniconazole, Chlorophyll, Endogenous hormones, Photosynthetic rate, Pathway

## Abstract

**Background:**

Duckweed is a novel aquatic bioenergy crop that is found ubiquitously throughout the world. Uniconazole plays an important role in improving crop production through the regulation of endogenous hormone levels. We found that a high quantity and quality of duckweed growth can be achieved by uniconazole application, although the mechanisms are unknown.

**Results:**

The fronds of *Landoltia punctata* were sprayed evenly with 800 mg/L uniconazole. The dry weight following treatment increased by 10% compared to the controls at 240 h. Endogenous cytokinin (CK) and abscisic acid (ABA) content both increased compared to the control, while the level of gibberellins (GAs) decreased. Additionally, gene expression profiling results showed that the expression of transcripts encoding key enzymes involved in endogenous CK and ABA biosynthesis were up-regulated, while the transcripts of key enzymes for GAs biosynthesis were down-regulated. On the other hand, chlorophyll a and chlorophyll b contents were both increased compared with the control. Moreover, the net photosynthetic rate was elevated to 25.6 μmol CO_2_/m^2^/s compared with the control value of 22.05 μmol CO_2_/m^2^/s. Importantly, the expression of some chlorophyll biosynthesis-related transcripts was up-regulated.

**Conclusion:**

Uniconazole treatment altered endogenous hormone levels and enhanced chlorophyll content and net photosynthetic rate in duckweed by regulating key enzymes involved in endogenous hormone and chlorophyll biosynthesis. The alterations of endogenous hormones and the increase of chlorophyll and photosynthetic rate data support the increase of biomass and starch accumulation.

**Electronic supplementary material:**

The online version of this article (doi:10.1186/s13068-015-0246-7) contains supplementary material, which is available to authorized users.

## Background

Duckweed, a novel aquatic bioenergy crop, has attracted widespread attention in recent years [1,2]. Duckweed is the simplest and smallest flowering aquatic plant that not only has a longer growing period but also grows faster than most other plants including field crops [3,4]. The growth rate of duckweed can reach 12 g/m^2^/day dry weight [5], and its yield has been documented to reach 55 tons/hectare/year dry weight in warm regions [5]. Depending on the duckweed species and the growing conditions applied, the starch content of duckweed ranges from 3% to 75% [6,7]. Duckweed also displays a significant ability to remove nutrients from sewage and has been applied for the treatment of municipal and industrial wastewater [8,9]. Moreover, its high starch accumulation and low lignin content are characteristics that are useful for bioethanol production [10]. Duckweed has been successfully converted to ethanol [11-13] and butanol [14] in recent years, indicating that it would be a good candidate feedstock for bioenergy production.

The use of plant growth regulators is a common and efficient method to manipulate plant growth and yield. Reports have shown that ‘Atlantic’ potato yields increase 15% after the application of a mixture of gibberellic acid, indole-butyric acid, and kinetin [15]. The foliar treatment of soybean plants with uniconazole increased the total biomass and seed yield by 8% and 18%, respectively, compared to the control [16]. Thus, we used plant growth regulator to improve the starch and biomass yield of duckweed for bioethanol utilization. We systematically screened more than 20 types of plant growth regulators, including auxins, cytokinins (CKs), abscisic acid (ABA), and gibberellins (GAs). One of the main findings of the screening was that uniconazole could simultaneously increase starch and biomass accumulation of duckweed under eutrophic conditions. Uniconazole, a plant growth retardant, has been extensively applied in plants to increase tolerance and improve quality [17-19]. It has potential advantages and various functions, including enhancing plant dry weight [20,21], increasing carotenoid and chlorophyll content in wheat and cucumber [22,23], and regulating endogenous hormones levels [24,25]. However, the mechanisms by which uniconazole regulates endogenous hormone and chlorophyll content is still unclear.

Next-generation sequencing (NGS) technology [26,27] is significant for the study of non-model plants. An increasing number of large-scale, high-throughput gene expression studies, as well as the sequencing of entire transcriptomes and genomes, are being conducted in some plants due to the advent of NGS technology. It is difficult to research metabolic pathways in non-model plants using conventional biological techniques. However, NGS technologies can detect millions of transcripts and can be used for the study of metabolic pathways. These techniques have been applied to investigations in some non-model plants [28,29], especially for successful study using transcriptome analysis to analyze metabolic pathways in duckweed [30]. In this study, we analyzed the chlorophyll and endogenous hormone biosynthetic pathways using NGS technology combined with biochemical assays and net photosynthetic rates to understand the process of biomass and starch accumulation. This analysis provides a large amount of information for further development of duckweed as a bioenergy crop.

## Results

### Impact of uniconazole on biomass accumulation of *L. punctata*

*L. punctata* 0202, originally collected from Sichuan, China, is a widely distributed duckweed species with great potential for starch accumulation. In this study, the fronds were sampled at 0, 1, 2, 3, 5, 7, 12, 24, 48, 72, 120, 168, and 240h time points post-treatment to measure dry weight and production of endogenous hormones and chlorophyll.

**Figure 1:**
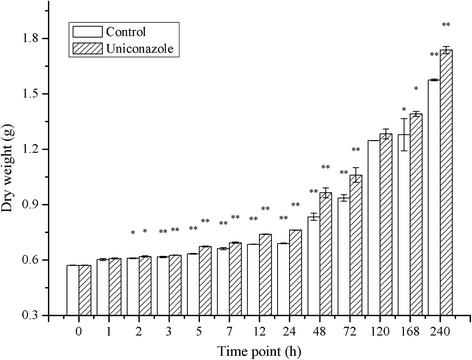
**The dry weight accumulation of** ***L. punctata*** **.** Each data point represents the mean of triplicate values; error bars indicate the standard deviation. * and ** indicate significant differences at the *P* = 0.05 and *P* = 0.01 probability level, respectively.

The results showed that there is little difference in dry weight between the 24 h control and treated samples. However, at 48 h, the dry weight of the treated sample was higher than that of the control. The dry weight was 0.834 and 0.965 g in control and treated samples, respectively (*P* < 0.05). At 240 h, the dry weight increased to 1.576 and 1.737 g, respectively, with an increase of 10% (*P* < 0.05) compared to that of the control (Figure 1). Moreover, uniconazole treatment also promoted starch accumulation. The dry weight of starch was 48.01% in the treated group and 15.7% in the control group after 240 h in culture. Please see our accompanying report for more details (accompanying report).

### Alteration of endogenous abscisic acid, cytokinin, and gibberellins levels in *L. punctata*

**Figure 2:**
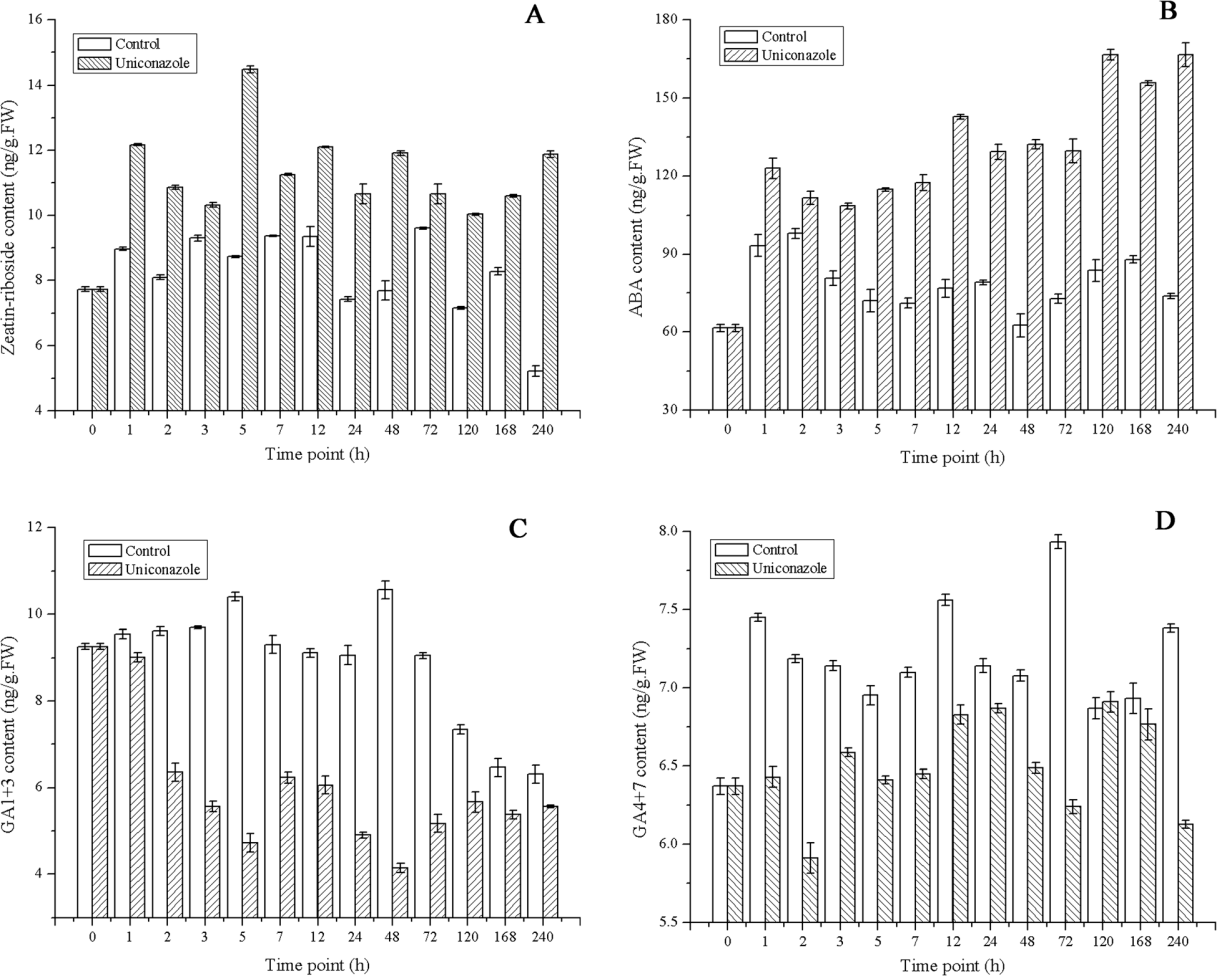
**Effect of uniconazole treatment on endogenous ZR, ABA, and GA levels in duckweed. (A)** The ZR content in fronds after uniconazole application. **(B)** The ABA content in fronds after uniconazole application. **(C)** The GA_1+3_ content in fronds after uniconazole application. **(D)** The GA_4+7_ content in fronds after uniconazole application. FW, fresh weight. Values are given as the mean ± SD of three experiments for each group.

It has been reported that uniconazole inhibits GAs synthesis, increase CKs synthesis, and transiently increases ABA content [31,32]. To investigate whether endogenous hormones are involved in the response to uniconazole application, the content of CKs, ABA, and GAs content was measured. As described in Figure 2, one type of CKs, Zeatin-riboside (ZR), was detected. ZR content increased from 7.73 to 11.87 ng/g (FW (fresh weight)) after uniconazole application but decreased to 5.22 ng/g (FW) in the control sample. The ABA content increased from 61.47 ng/g (FW) to 166.53 ng/g (FW) after uniconazole application, while there was little change in the control. GA_1+3_ content decreased from 9.25 to 5.57 ng/g (FW) following treatment compared to 6.3 ng/g (FW) in the control after 240 h of growth. GA_4+7_ levels decreased from 6.37 to 6.13 ng/g (FW) following treatment and increased to 7.38 ng/g (FW) in the control (Figure 2 C,D).

### Effect of uniconazole on *L. punctata* chlorophyll content and photosynthesis

**Figure 3:**
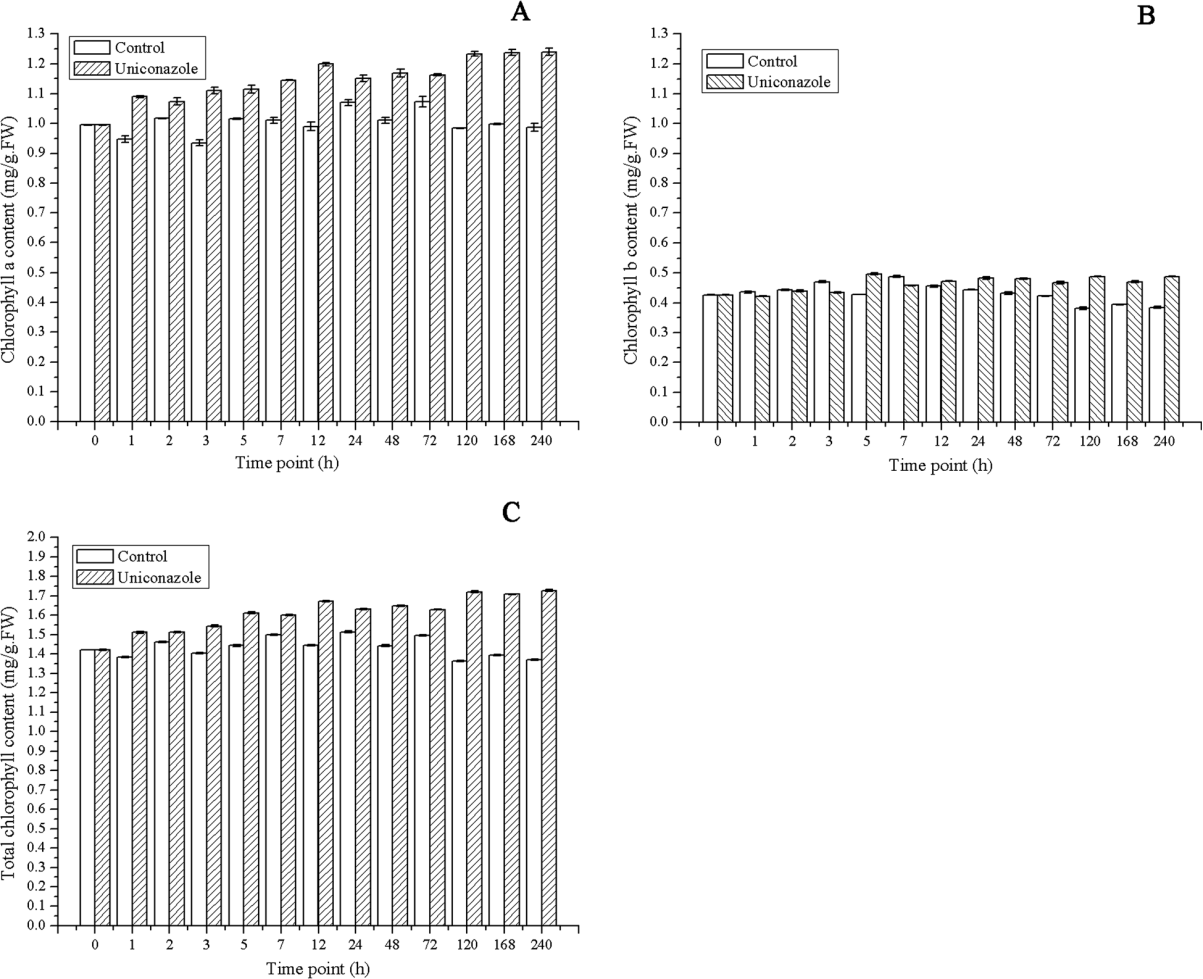
**Chlorophyll a and chlorophyll b content of** ***L. punctata.*** **(A)** Chlorophyll a content. **(B)** Chlorophyll b content. **(C)** Total chlorophyll content. Values are given as the mean ± SD of three experiments for each group.

As shown in Figure 3, chlorophyll a content increased from the initial value of 0.998 to 1.239 mg/g (FW) following treatment and decreased slightly to 0.987 mg/g (FW) in the control sample. Similarly, chlorophyll b content increased from the initial value of 0.426 to 0.488 mg/g (FW) following treatment and decreased mg/g (FW) to 0.384 mg/g (FW) in the control sample.

**Figure 4:**
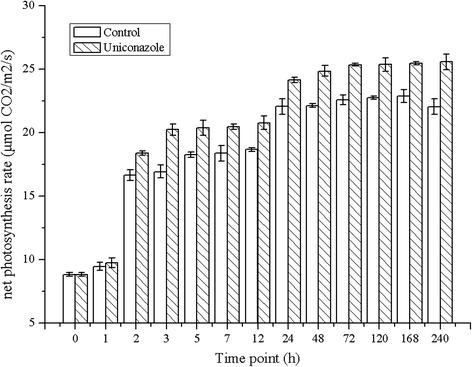
**Effects of uniconazole treatment on the net photosynthesis rate of duckweed.** All data are presented as the mean of triplicate measurements ± standard deviation.

To analyze the impact of uniconazole treatment on photosynthesis and respiration, net photosynthesis rates were measured, as shown in Figure 4. The net photosynthesis rate increased from 8.83 μmol CO_2_/m^2^/s to 16.94 and 20.24 μmol CO_2_/m^2^/s at 3 h for the control and treatment groups, respectively. During this time period, uniconazole treatment remarkably increased the net photosynthesis rate of duckweed by 19.5% compared to the control. Finally, at the 240-h time point, the net photosynthesis rate of duckweed reached 22.05 and 25.6 μmol CO_2_/m^2^/s, respectively. Uniconazole treatment increased the net photosynthesis rate of duckweed by 16.2% compared with the control.

### Sequencing, *de novo* assembly, and functional annotation of the *L. punctata* transcriptome

To investigate the genome-wide expression patterns of uniconazole treated *L. punctata*, samples collected at the 0, 2, 5, 72, and 240h time points were used for RNA-Seq analysis. In total, 48,315,010, 48,390,098, 48,623,932, 48,282,456, and 48,248,454 paired end (PE) 90 bp reads were obtained for each sample, respectively, corresponding to 21.77 G bp data (Table 1). These sequence reads were deposited in NCBI’s Sequence Read Archive (SRA) database (http://www.ncbi.nlm.nih.gov/Traces/sra/sra.cgi?) under the accession number PRJNA242298. All reads were pooled together with the 118,754,706 PE reads obtained from nutrient starvation-treated *L. punctata* and *de novo* assembled using Trinity (v2012-06-08) [33]. A total of 140,432 contigs with lengths ≥200 bp were assembled. The average length, the N50 length, and the max length of these contigs were 1,131, 2,197, and 18,144 bp, respectively. There were 133 and 50,008 contigs longer than 10,000 and 1,000 bp, respectively (Table 1). All assembled sequences were deposited in NCBI’s Transcriptome Shotgun Assembly (TSA) database (http://www.ncbi.nlm.nih.gov/genbank/tsa/) under the accession number PRJNA242298. Of the 140,432 contigs, 91,303 (65.0%) had annotation information (Additional file 1: Table S1). For contigs with lengths ≥1,000 bp, 94.7% had BLASTX hits. For contigs with lengths ≥600 bp, this percentage was 87.8%. These results indicated that most of the contigs were protein-encoding transcripts. To assess the final assembly, we calculated the ratios of long CDS-containing transcripts to the total corresponding length contigs (A contig (from contiguous) is a set of overlapping DNA segments that together represent a consensus region of DNA [34]). There were 44,272 contigs with lengths ≥1200 bp, and 23,569 (53.2%) contained long-CDS with lengths ≥1,200 bp. For contigs with lengths ≥900 or 600 bp, this ratio was 71.1% (37,879 of 53,265) and 83.7% (55,356 of 66,138), respectively. For contigs with lengths ≥1,200 bp, 85.6% contained long CDS with lengths ≥900 bp.

**Table 1:** **Assembly statistics of the** ***L. punctata*** **transcriptome**

**Items**	**Characteristics**
PE read number	241,859,950
Contig number	140,432
Contig ≥10,000 bp	133
Contig ≥2,000 bp	25,641
Contigs >1,000 bp	50,008
Average length (bp)	1,131
Max length (bp)	18,144
N50 length (bp)	2,197
Total length (bp)	158,793,737

PE reads from five *L. punctata* samples were pooled together and assembled using Trinity (v2012-06-08). Statistics were conducted by common perl scripts.

### Expression analysis of transcripts encoding key enzymes involved in CK, ABA, and GA biosynthesis

**Figure 5:**
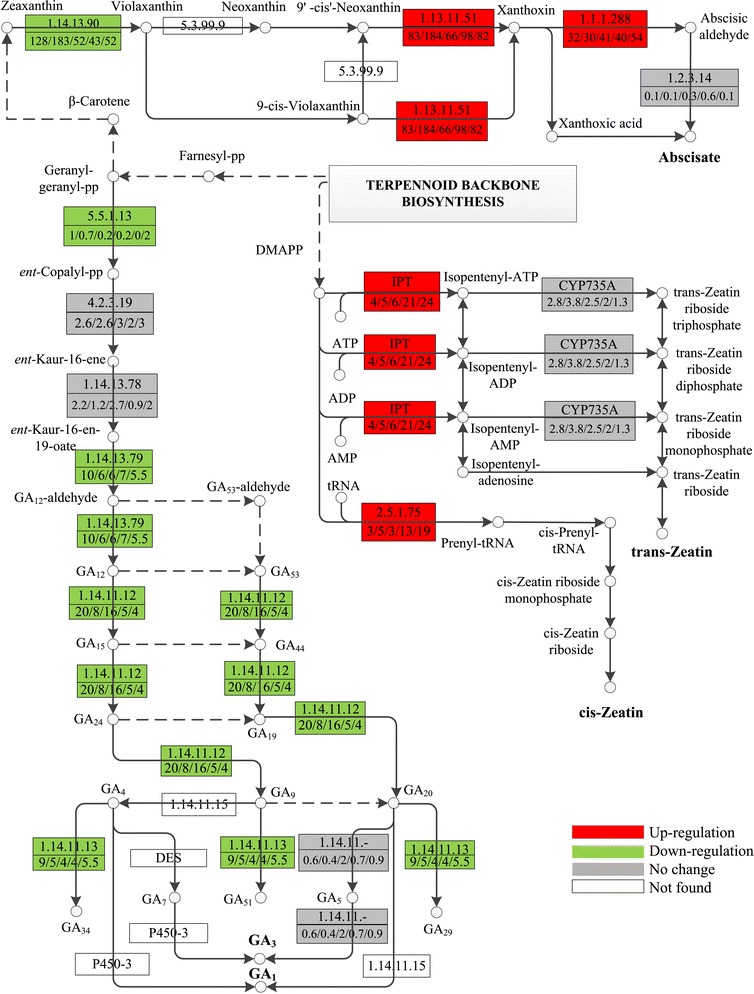
**Expression patterns of Zeatin, abscisic acid (ABA), and gibberellins (GAs) metabolism related transcripts.** Expression alterations of Zeatin, ABA, and GA metabolism-related transcripts are displayed in the simplified metabolism pathway. Red boxes indicate the up-regulated enzymes, green means down-regulated enzymes, gray means no significant difference were observed, and white means this enzyme was not found in this study. The numbers in the upper half of the boxes correspond to the EC numbers and the numbers in the lower half, separated by slashes, correspond to the expression levels of these enzymes shown in FPKM at 0, 2, 5, 72, and 240 h, respectively. 2.5.1.27, adenylate isopentenyltransferase; IPT, adenylate isopentenyltransferase; CYP735A, cytokinin trans-hydroxylase; 1.14.13.90, zeaxanthin epoxidase; 1.13.11.51, 9-cis-epoxycarotenoid dioxygenase; 1.2.3.14, abscisic-aldehyde oxidase; 1.1.1.288, xanthoxin dehydrogenase; 5.5.1.13, ent-copalyl diphosphate synthase; 4.2.3.19, ent-kaurene synthase; 1.14.13.78, ent-kaurene oxidase; 1.14.13.79, ent-kaurenoic acid hydroxylase; 1.14.11.12, gibberellin 20-oxidase; 1.14.11.13, gibberellin 2-oxidase.

Expression pattern analysis showed that uniconazole application changed the expression of most transcripts encoding key enzymes involved in CK, ABA, and GA biosynthesis (Additional file 2: Table S2). The biosynthesis of CK includes two types of pathway: tRNA involved in the cytokinin biosynthesis pathway and the AMP-, ADP-, and ATP-mediated cytokinin biosynthesis pathways (Figure 5). Adenylate isopentenyltransferase (IPT EC: 2.5.1.27) is the rate-limiting enzyme involved in the AMP-, ADP-, and ATP-mediated cytokinin biosynthesis pathway [35]. IPT transcripts (comp16957_c0_seq2) increased from 4 to 24.1 FPKM at 240 h. The tRNA dimethylallyltransferase (EC: 2.5.1.75) transcript, which is another important enzyme involved in the cytokinin biosynthesis pathway, increased from 2.9 FPKM at 0 h to 5.0 FPKM at 2 h and finally reached 19 FPKM at 240 h (comp16957_c0_seq1).

The expressions of transcripts encoding the rate-limiting enzymes for ABA biosynthesis (9-cisepoxycarotenoid dioxygenase (NCED) and aldehyde oxidase (AO)) were up-regulated by uniconazole treatment. The expression of NCED increased from 83.64 to 184.4 FPKM at 2 h (comp41219_c0_seq1). The expression of AO increased from 32.32 to 54.08 FPKM at 240 h (comp82056_c0_seq1).

The transcripts of enzymes involved in gibberellins (GAs) biosynthesis are described in Figure 5. Ent-copalyl diphosphate synthase (EC: 5.5.1.13) catalyzed the first step of formation of diterpene cyclization, which plays an important biological role during the GA biosynthetic pathway. Expression analysis showed that ent-copalyl diphosphate synthase (comp34724_c0_seq1) decreased from 1.03 to 0.21 FPKM at 240 h after uniconazole treatment. The transcript of ent-kaurenoic acid hydroxylase (EC:1.14.13.79, comp17707_c0_seq1), which is involved in the catalytic reaction for GA biosynthesis, also decreased from 10.35 to 5.51 FPKM at 240 h following treatment with uniconazole. Gibberellin 20-oxidase (EC: 1.14.11.12) is an important regulatory enzyme for GA biosynthesis. Data analysis exhibited decreased expression levels of gibberellin 20-oxidase (from 20 to 4 FPKM at 240 h (comp36626_c0_seq1)).

### Expression analysis of chlorophyll synthesis-related transcripts

**Figure 6:**
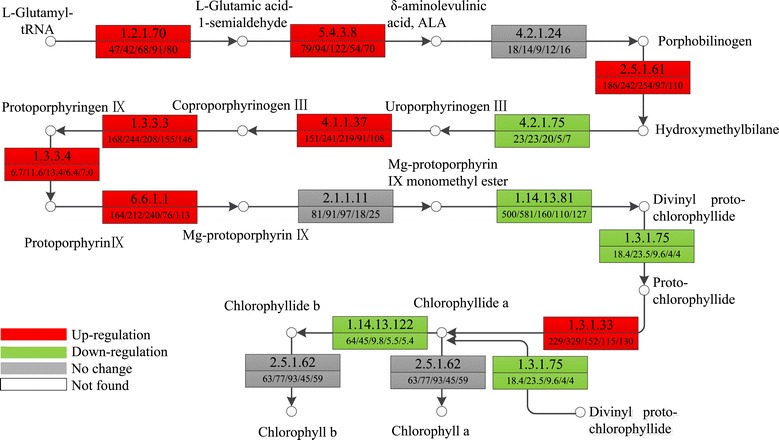
**Expression patterns of chlorophyll metabolism related transcripts.** Expression alterations of chlorophyll metabolism-related transcripts are displayed in the chlorophyll biosynthesis pathway. Red boxes indicate up-regulated enzymes, green means down-regulated enzymes, gray means no significant difference was observed, and white means this enzyme was not found in this study. The numbers in the upper half of the boxes correspond to the EC numbers and the numbers in the lower half, separated by slashes, correspond to the expression levels of these enzymes shown in FPKM at 0, 2, 5, 72, and 240 h, respectively. 1.2.1.70, glutamyl-tRNA reductase; 5.4.3.8, glutamate-1-semialdehyde 2,1-aminomutase; 4.2.1.24, porphobilinogen synthase; 2.5.1.61, hydroxymethylbilane synthase; 4.2.1.75, uroporphyrinogen-III synthase; 4.1.1.37, uroporphyrinogen decarboxylase; 1.3.3.3, coproporphyrinogen III oxidase; 1.3.3.4, protoporphyrinogen oxidase; 6.6.1.1, magnesium chelatase subunit H,D,I; 2.1.1.11, magnesium-protoporphyrin o-methyltransferase; 1.14.13.81, magnesium-protoporphyrin IX monomethyl ester (oxidative) cyclase; 1.3.1.75, divinyl chlorophyllide a 8-vinyl-reductase; 1.3.1.33, protochlorophyllide reductase; 2.5.1.62, chlorophyll synthase; 1.14.13.122, chlorophyllide a oxygenase.

Chlorophyll (Chl) is an important photosynthetic pigment in the chloroplast of plants, and the metabolism of chlorophyll is an important factor in determining crop yield. We have found that chlorophyll content increased after uniconazole application. To investigate whether chlorophyll synthesis-related genes were involved in the increase in chlorophyll content, we further studied expression patterns of regulatory enzymes involved in chlorophyll biosynthesis (Figure 6). There are 15 enzymes required for chlorophyll biosynthesis from glutamyl-tRNA to chlorophyll b [36]. Glutamyl-tRNA reductase (EC: 1.2.1.70) is the rate-limiting enzyme in the synthesis of δ-aminolevulinic acid (ALA) and determines the total flow rate of the chlorophyll synthesis pathway [37]. Expression analysis demonstrated that expression levels were up-regulated from 46.9 to 91 FPKM (comp16336_c0_seq1). Mg-chelatase (EC: 6.6.1.1) is another key enzyme involved in chlorophyll synthesis. Transcripts encoding Mg-chelatase (comp41026_c0_seq1) exhibited expression levels of 164 FPKM at 0 h and increased to 212 FPKM at 2 h and 240.7 FPKM at 5 h. NADPH-protochlorophyllide oxidoreductase (POR, EC: 1.3.1.33) catalyzes protochlorophyllide to synthesize chlorophyllide a, which is a key enzyme involved in chlorophyll synthesis. Expression analysis showed that POR (comp40956_c0_seq1) was also up-regulated, with expression levels increased from 229.54 to 329.28 FPKM (Additional file 3: Table S3). To further explain the increased photosynthesis rate, we analyzed the gene expression of chlorophyll binding proteins. The expressions of transcripts encoding chlorophyll binding proteins were up-regulated by uniconazole treatment, the expression of chlorophyll binding proteins (comp40465_c0_seq2) increased from 1,071 to 4,287, 1,399, and 1,066 FPKM at 2, 5, and 72 h, respectively. The expression of chlorophyll-binding proteins decreased to 816.43 FPKM at 240 h.

## Discussion

### Uniconazole affects the biosynthesis of endogenous CKs, ABA, and GA

CKs, ABA, and GAs, as members of the plant terpenoids, are three important types of broad-spectrum plant growth regulators that are widely used to enhance plant development and metabolism. Numerous studies have focused on changes in levels in plant hormones that regulate plant growth and development, such as ABA, GAs, ZR, and IAA [38-41]. Several studies have focused on the expression levels of genes involved in hormone biosynthesis; gene chip technology is often used to analyze the expression of genes during the growth and development process [42]. Capron [43] used the Nimble Gen microarray to study gene expression during the wheat grain filling stage and discovered hormone-related gene expression during the early and late stages of the grain.

Endogenous hormone levels and transcriptomics data were integrated to elucidate the process of endogenous hormone changes after uniconazole application. The biochemical assays showed that the ABA and ZR contents increased after uniconazole application. The expression patterns of key enzyme-encoding transcripts involved in endogenous ZR, ABA, and GA biosynthesis further supported the change of hormones levels described above. Transcriptomics data showed that transcripts of enzymes involved in ABA and ZR synthesis were up-regulated, while enzymes involved in GA synthesis were down-regulated. The expression levels of transcripts encoding IPT were up-regulated from 4 to 24.1 FPKM at 240 h. Moreover, 9-cisepoxycarotenoid dioxygenase (NCED), which is involved in ABA biosynthesis, was up-regulated from 83.64 to 184.4 FPKM at 2 h. These data were consistent with the increases in ZR and ABA levels detected with the biochemistry assay. Additionally, the expression levels of transcripts encoding gibberellin 20-oxidase were down-regulated from 11.49 to 0.61 FPKM at 240 h, supporting the decreased content of GA observed in the biochemical assay.

CK regulates numerous growth and developmental processes by regulating cell division and cell differentiation [44,45]. Moreover, reports have shown that the increase of CKs plays an important role in regulating grain filling pattern and consequently elevated starch accumulation. In this study, the increase of CK can promote the biomass accumulation of duckweed by regulating chlorophyll cell division and cell differentiation. Hormones can mediate changes directly by binding to intracellular hormone receptors and modulating gene transcription or indirectly by binding to cell surface receptors and stimulating signaling pathways. In the signaling pathway of CK [45,46], CK is perceived by the cytokinin receptors AHK2, AHK3, and AHK4; this activates a multistep phosphorelay. CK binding to AHKs activates autophosphorylation (*P*) via AHPs in the cytoplasm. The AHPs translocate into the nucleus to transfer the phosphoryl group (*P*) to type-B ARR. Then phosphorylation of type-B ARRs interact with the promoter of STAY-GREEN (SGR); this interaction was assayed to determine whether the cytokinin signaling pathway interacted with a key step in chlorophyll degradation within the chloroplast [47] (data not shown). The up-regulated expression of transcripts encoding key enzymes involved in endogenous CK also supported the increased content of chlorophyll.

The plant hormone ABA is involved in the regulation of many developmental processes in plants, including the induction of seed dormancy and stimulation of starch accumulation [48]. Reports have shown that ABA can up-regulate AGPase gene transcription in rice suspension cells [49], suppress the expression of genes encoding amylases and proteases [50], and promote starch biosynthesis [51]. The most important key enzymes involved in starch synthesis are ADP-glucose pyrophosphorylase (AGPase), soluble starch synthase (SSS), and starch-branching enzyme (SBE). The increase of ABA levels may activate starch accumulation in duckweed by regulating key enzymes involved in starch biosynthesis. In the upstream of the ABA signaling pathway, the PYLs act as ABA receptors [52]. In the presence of ABA, ABA combines with intracellular PYL and PP2C to form an ABA-PYL-PP2C complex. This complex inhibits the activity of PP2C in an ABA-dependent manner and activates autophosphorylation (*P*) of SnRK2s. Then, the phosphoryl group (*P*) from SnRK2s is transferred to abscisic acid insensitive 4 (ABI4), thereby inducing ADP-glucose pyrophosphorylase subunit AGPLs (*ApL3*) gene expression. The increase expression of *ApL3* may finally result in the starch accumulation (data not shown).

The change in endogenous hormones levels was consistent with the biomass and starch accumulation described above. Moreover, we found that starch accumulation in duckweed was accompanied by alteration of endogenous hormone levels. The relationship between endogenous hormones and starch accumulation will be discussed in the accompanying report (accompanying report).

### The impact of uniconazole on chlorophyll biosynthesis

Chlorophyll (Chl) is an important photosynthetic pigment in the chloroplast of plants that performs the essential processes of harvesting light energy in the antenna systems [53]. The metabolism of chlorophyll is an important factor in determining the photosynthetic rate and affects crop yield [54]. To date, numerous studies have investigated the relationship between the development and metabolism of plants and photosynthesis. Chlorophyll content was assayed in most studies [55]. Other studies have focused on regulatory factors involved in chlorophyll synthesis, such as transcription factors and regulatory proteins [56,57]. Song [58] used microarray hybridization to identify genes involved in photosynthesis and chlorophyll synthesis in response to heat stress, but no metabolic pathways were mentioned. In this study, a genome-wide transcriptomic analysis method was used to investigate the metabolism of key enzymes involved in the chlorophyll biosynthesis pathway by us.

In this study, uniconazole enhanced the chlorophyll content, up-regulated the expression of key enzymes involved in the chlorophyll biosynthesis pathway, and increased the net photosynthesis rate. The data from three different lines of evidence were analyzed and compared. The results of the biochemical assay showed that chlorophyll a content increased from an initial value of 0.998 to 1.239 mg/g (FW) at 240 h, and chlorophyll b content increased from a value of 0.426 to 0.488 mg/g (FW) at 240 h. Chlorophyll a and chlorophyll b contents increased by 25.6% and 27%, respectively, compared to that of the control. The increased chlorophyll content was further verified by the expression pattern of transcripts encoding key enzymes involved in chlorophyll biosynthesis. Transcriptomic analysis revealed that the expression of three key enzymes involved in the synthesis of chlorophyll were up-regulated. The expression levels of transcripts encoding glutamyl-tRNA reductase (EC: 1.2.1.70) were up-regulated from 46.9 to 80 FPKM at 240 h. Transcripts encoding Mg-chelatase were up-regulated from 164 to 240.7 FPKM at 5 h. Chlorophyll-binding proteins (CBPs) constitute a large family of proteins with diverse functions in both light-harvesting and photoprotection. The regulation of the CBPs expression is considered to be one of the important mechanisms for plants to modulate chloroplast functions. Transcriptome results showed that the expression of chlorophyll-binding proteins were up-regulated after uniconazole application, which also supported the increased photosynthesis rate. Importantly, the enhanced content of chlorophyll was further supported by the net photosynthesis rate data. The net photosynthesis rate increased from 8.83 μmol CO_2_/m^2^/s at 0 h to 22.05 and 25.6 μmol CO_2_/m^2^/s at 240 h in the control and treatment groups, respectively. Uniconazole treatment increased the duckweed net photosynthesis rate by 16.2% compared to the control. The increase of net photosynthesis rate data supported the biomass and starch accumulation of duckweed.

## Conclusions

Uniconazole treatment enhanced chlorophyll content and net photosynthetic rate and altered endogenous hormone levels in duckweed by regulating the expression of key enzymes involved in chlorophyll and endogenous hormones biosynthesis. These physiological, biochemical, and transcriptomics data supported the increase dry weight and starch accumulation of duckweed. This study provides a comprehensive transcriptome and genome-wide gene expression profile of *L. punctata* after uniconazole application. These results provide valuable information that paves the way for further molecular biological studies of plant hormone applications in duckweed.

## Materials and methods

### Duckweed cultivation and uniconazole treatments

*Landoltia punctata* 0202 was originally collected from Sichuan Province, China. It was cultivated in standard 1/6 Hoagland E+ solution (total *N* = 58.3 mg/L, *P* = 25.8 mg/L) [59] culture for 3 days under a 16/8-h day/night photoperiod, with a light intensity of 130 μmol/m^2^/s and a temperature of 25°C/15°C at day/night. Then, 6 g fronds were transferred into 1,000 mL 1/6 Hoagland E+ culture plastic containers (23 × 14 × 4.5 cm) for further cultivation over a period of 10 days. Uniconazole powder (S: *R* = 79:21) was manufactured by Sumitomo Chemical (Osaka, Japan). The concentration of uniconazole used in this study was 800 mg/L. To investigate the effect of uniconazole on *L. punctata*, a 5 mL solution of 800 mg/L uniconazole was sprayed evenly on the surface of the fronds. Controls were sprayed with 5 mL water containing 10% methanol. The experiments were carried out with three replicates. Thirteen different time points, including 0, 1, 2, 3, 5, 7, 12, 24, 48, 72, 120, 168, and 240 h after fronds were cultured in solution, were chosen for composition and enzymatic activity assays. For each time point, the fronds were collected from three culture plastic containers. Samples collected at 0, 2, 5, 72, and 240 h were frozen in liquid nitrogen immediately for the RNA-Seq study.

### Material composition

To measure the fresh weight (FW), the fronds were measured with a balance using Bergmann’s method [60]. To measure the dry weight (DW), the samples were dried at 60°C until the weight was constant.

The extraction, purification, and determination of endogenous levels of ZR, ABA, and GAs (GA_1+3_, GA_4+7_) by an indirect ELISA technique were performed as described by Wang [61] and Yang [62]. Chlorophyll content was analyzed according to the method described by Aron [63]. The net photosynthetic rate was determined by Li-6400xt (LI-COR, Inc., St. Lincoln, NE, USA) using the whole chamber (6400–17) and the RGB light source (6400–18).

### RNA extraction and cDNA fragment library construction

Five *L. punctata* samples were collected at 0, 2, 5, 72, and 240 h after treatment with uniconazole. For each sample, total RNA was extracted from 200-mg fronds using the OMEGATM Plant DNA/RNA Kit (OMEGA, New York, NY, USA). Genomic DNA was digested using DNase I (Fermentas, Waltham, MA, USA) according to the manufacturer’s instructions. RNA concentration, OD260/280, OD260/230, 28S/18S, and RNA integrity number (RIN) were measured with an Agilent 2100 Bioanalyzer (Agilent Technologies Inc., Santa Clara, CA, USA) or NanoDrop (Thermo Fisher Scientific, Waltham, MA, USA). Qualified total RNA extracted from each sample was submitted to the Beijing Genomics Institute (BGI)-Shenzhen, Shenzhen, China [http://www.genomics.cn] for RNA sequencing by Illumina HiSeq 2000 (Illumina, San Diego, CA, USA).

The poly (A) + RNAs were purified using poly-T oligoattached magnetic beads and eluted with Tris–HCl under heating condition. mRNAs were mixed with fragmentation media and then fragmented. Fragmented mRNAs were copied into first strand cDNA using reverse transcriptase and random primers. This is followed by second strand cDNA synthesis using DNA polymerase I and RNaseH. The ends of these dscDNAs were repaired by adding a single ‘A’ base and then Illumina adapters (Illumina, San Diego, CA, USA) ligated to the repaired ends. cDNAs fragment, 200 bp, were purified from a gel and used for further template enrichment by PCR with two primers that anneal to the ends of the adapters to construct a fragmented cDNAs library. Quality control analysis was performed by Agilent 2100 Bioanalyzer. Library quality control analysis was performed with an Agilent 2100 Bio-analyzer.

### RNA sequencing and paired end reads assembly

The validated 200-bp fragments from the cDNA libraries were submitted to Illumina HiSeq 2000 platform for PE RNA sequencing. PE read sequencing quality was assessed by fastqc [http://www.bioinformatics.bbsrc.ac.uk/projects/fastqc/] and then *de novo* assembled using Trinity (v2012-06-08) [33] under default parameter choices. All PE reads were used to align back to these assembled sequences using the Bowtie2 (v2.0.0-beta5) program [64]. Accordingly, the read align rate was calculated. Length distribution analysis was performed with common perl scripts to calculate the N50 number, average length, and max length. The best candidate open reading frame (ORF) was predicted using perl scripts in the Trinity package (v2012-06-08) [33].

### Functional annotation and cluster

All contigs assembled by Trinity (v2012-06-08) were submitted to Blast2GO [65,66] for functional annotation. A BLASTX similarity search performed against the NR database [http://www.ncbi.nlm.nih.gov/] by Blast2GO with a threshold *E*-value <10^3^. Enzyme codes were extracted and Kyoto Encyclopedia of Genes and Genomes (KEGG) pathways were retrieved from the KEGG web server [http://www.genome.jp/kegg/]. A KEGG pathway cluster was conducted using common perl scripts.

### Expression pattern analysis and verification

To analyze the expression level of each transcript at different time points following treatment with uniconazole, all PE reads for each sample were used for mapping analysis with perl scripts in the Trinity package (v2012-06-08) under default parameter choices. The expression value of each transcript was calculated and normalized according to the RESM-based algorithm using the perl scripts in the Trinity (v2012-06-08) package to obtain FPKM values. *P*-values and log_2_ fold change (log_2_FC) were calculated; then, significantly differentially expressed transcripts (DETs) between two sample sets were identified with *P* values ≤0.05 and log_2_FC ≥1. Hypergeometric tests based on KEGG annotation were performed for each DETs group identified between two sample sets using common R scripts to extract the enriched KEGG pathway.

### Calculations and statistics

Each data point represents the results of three sample experiments; the results are presented as means ± standard error in the figures.

## Additional files

Additional file 1: Table S1. Sequence annotations of *L. punctata* transcripts and the gene expression profiling of five samples. Additional file 2: Table S2. Expression levels of several plant hormone metabolism pathway related genes. Additional file 3: Table S3. Expression levels of some chlorophyll biosynthesis related genes.

## Abbreviations

ABA: abscisic acid
CKs: cytokinins
DET: differentially expressed transcript
DW: dry weight
EC: enzyme codes
FPKM: fragments per kilobase of transcripts per million mapped fragments
FW: fresh weight
GAs: gibberellins
KEGG: Kyoto Encyclopedia of Genes and Genomes
log_2_FC: log_2_ fold change
NGS: next-generation sequencing
PE: paired end
